# Formation of Free-Standing Inverse Opals with Gradient Pores

**DOI:** 10.3390/nano10101923

**Published:** 2020-09-26

**Authors:** Pei-Sung Hung, Chen-Hong Liao, Bo-Han Huang, Wei-An Chung, Shou-Yi Chang, Pu-Wei Wu

**Affiliations:** 1 Department of Materials Science and Engineering, National Chiao Tung University, Hsinchu 300, Taiwan; hps198810@hotmail.com (P.-S.H.); martin.liao1203@gmail.com (C.-H.L.); justin1124.mse96@g2.nctu.edu.tw (B.-H.H.); fsmuniverse42@gmail.com (W.-A.C.); 2 Department of Materials Science and Engineering, National Tsing Hua University, Hsinchu 300, Taiwan; changsy@mx.nthu.edu.tw

**Keywords:** colloidal crystals, inverse opals, gradient pores, electrophoresis, self-assembly, mechanical properties

## Abstract

We demonstrate the fabrication of free-standing inverse opals with gradient pores via a combination of electrophoresis and electroplating techniques. Our processing scheme starts with the preparation of multilayer colloidal crystals by conducting sequential electrophoresis with polystyrene (PS) microspheres in different sizes (300, 600, and 1000 nm). The critical factors affecting the stacking of individual colloidal crystals are discussed and relevant electrophoresis parameters are identified so the larger PS microspheres are assembled successively atop of smaller ones in an orderly manner. In total, we construct multilayer colloidal crystals with vertical stacking of microspheres in 300/600, 300/1000, and 300/600/1000 nm sequences. The inverse opals with gradient pores are produced by galvanostatic plating of Ni, followed by the selective removal of colloidal template. Images from scanning electron microscopy exhibit ideal multilayer close-packed structures with well-defined boundaries among different layers. Results from porometer analysis reveal the size of bottlenecks consistent with those of interconnected pore channels from inverse opals of smallest PS microspheres. Mechanical properties determined by nanoindentation tests indicate significant improvements for multilayer inverse opals as compared to those of conventional single-layer inverse opals.

## 1. Introduction

The synthesis of materials with tailored porosity and pore size has attracted considerable attention for their promising potentials in electrocatalysis, sensing, scaffolds, and capacitors [1,2,3,4,5,6]. Among many porous materials studied, the inverse opals—also known as the 3D ordered macroporous materials—have received the most scrutiny because their macropores are arranged in a honeycombed structure with 12 pore channels connecting neighboring macropores [7]. This unique configuration enables an excessive surface area with facile mass transport. In addition, the honeycombed structure is recognized to reveal improved mechanical properties as compared to alternative nanostructured counterparts. So far, inverse opals consisting of metals, oxides, and polymers in a wide range of pore sizes have been demonstrated in studies [8,9,10,11]. 

The fabrication of inverse opals starts with the construction of colloidal crystals serving as the template to allow the filling of selective materials into the interstitial voids among the close-packed microspheres, followed by the removal of the colloidal template. Therefore, the formation of ideal colloidal crystals is critical to ensure inverse opals with desired microstructures. Conventional approaches to prepare colloidal crystals entails the packing of monodispersive microspheres via spontaneous assembly routes including sedimentation, solvent evaporation, interface control, and spin coating [12,13,14,15,16]. In these demonstrations, the low yield and long processing time limits the potential use of self-assembly processes for device fabrication [17,18,19,20,21]. The resulting single-layer colloidal crystals adopt a polycrystalline structure whose grain sizes are relatively limited and for which crystallographic defects—such as vacancies, grain boundaries, and voids—are inevitable. These physical constraints are detrimental in fabricating inverse opals with desirable microstructures. Moreover, the demonstration of free-standing inverse opals is unlikely due to their poor uniformity and lack of mechanical strength. 

In contrast, electrophoresis is a known process to deposit oxide polymers and small molecules for coating purposes, and many industrial applications have been demonstrated [22,23,24,25,26]. Earlier, we adopted the electrophoresis technique to produce single-layer colloidal crystals with significantly reduced crystallographic defects [27,28]. After identifying relevant processing parameters, we were able to demonstrate colloidal crystals in either planar and cylindrical form with precise colloidal layers, and their surfaces were rather uniform [29,30]. This allowed us to fabricate single-layer inverse opals with ideal hexagonal honeycombs and controlled thickness [31,32]. In addition, we have explored their possible applications in both gas sensing and electrochemical sensing [33,34,35]. Due to their improved crystallinity, the inverse opals were able to be detached from the substrate, becoming essentially a 3D ordered macroporous membrane [35,36]. We recognized that this free-standing inverse opaline film provides new opportunities for applications in filtering and selective adsorption. Therefore, it is of particular interest for us to explore the processing possibility in fabricating free-standing multilayer inverse opals, or an inverse opaline membrane with gradient pores. To the best of our knowledge, this type of material has not been demonstrated in the literature yet. 

In this work, we used a sequential electrophoresis approach to prepare colloidal crystals consisting of microspheres in different sizes (multilayer colloidal crystals), and used them as the template to fabricate inverse opals with gradient pores. We conducted extensive experiments to validate the critical packing rules for the successive assembly of microspheres in a vertical layer-by-layer arrangement. Because of multilayer colloidal templates without defects, the resulting multilayer inverse opals were easily removed from the substrates, forming free-standing inverse opaline membranes. In nanoindentation tests, these multilayer inverse opals revealed improved mechanical properties compared with their single-layer counterparts. 

## 2. Materials and Methods

### 2.1. Synthesis of Polystyrene Microspheres in Different Sizes

The polystyrene (PS) microspheres in various sizes were used to construct the multilayer colloidal crystals and they were synthesized by a soap-free emulsion polymerization process. In general, 1000 mL DI water was deoxygenated by N_2_ at 65 °C for 12 h in a thermostat reactor. Subsequently, styrene (St, 99 wt %, Sigma-Aldrich, St. Louis, MO, USA), sodium 4-styrenesulfonate (NaSS, 90 wt %, Alfa Aesar, Haverhill, MA, USA), and potassium bicarbonate (KHCO_3_, 99 wt %, J.T. Baker, Phillipsburg, NJ, USA) were dissolved, and they were used as the monomers, comonomers, and buffer, respectively. The mixture underwent constant stirring for 15 min to form a homogeneous solution. Next, potassium persulfate (99 wt %, Sigma-Aldrich) was added to initiate the polymerization step, and the polymerization lasted 16 h at 65 °C. Afterward, the mixture was cooled at 60 °C to evaporate residual solvent to retrieve PS microspheres in dry powders. In this study, we synthesized PS microspheres in diameter of 300, 600, and 1000 nm, and their respective synthetic parameters are listed in Table 1.

### 2.2. Fabrication of Multilayer Colloidal Crystals and Their Inverse Opals

Prior to the electrophoresis process, the PS microspheres were suspended in anhydrous ethanol, and the suspension pH was adjusted by minute amount of 1 M HNO_3(aq)_ and 1 M NH_4_OH_(aq)_. To construct multilayer colloidal crystals, a sequential vertical electrophoresis technique was adopted in which a 4 × 4 cm^2^ Indium tin oxide (ITO) and a 10 × 10 cm^2^ stainless steel were used as the working and counter electrode, respectively. The electrophoretic cell contained 150 mL PS suspension and the distance between the electrodes was 1 cm. The sequential electrophoresis was conducted at 10 °C in separate PS suspensions with different PS microspheres, and the imposed electric field was adjusted accordingly. In total, we prepared multilayer colloidal crystals consisting of microspheres stacked in sequences of 300/600, 300/1000, and 300/600/1000 nm (from bottom to top). Afterwards, the multilayer colloidal crystals were retrieved and rinsed by DI water, followed by a mild heat treatment at 90 °C in air for 48–96 h. 

To fabricate inverse opals with gradient pores, we conducted the Ni electrodeposition using the Watt’s plating formula which contained 0.5 M NiSO_4_·6H_2_O (99.95 wt %, SHOWA, Gyoda, Saitama, Japan), 0.125 M NiCl_2_·6H_2_O (98 wt %, SHOWA), and 0.3 M H_3_BO_3_ (99.95 wt %, SHOWA) [36]. The working electrode was multilayer colloidal crystals in 4 × 4 cm^2^ and the counter electrode was a 6 × 6 cm^2^ Ni plate. The electroplating was carried out in a galvanostatic mode of 2.5 mA cm^−2^ for 90–210 min at 25 °C. Afterward, the sample was rinsed with DI water and dried in an oven in air at 50 °C. Next, the sample was immersed in toluene (99.5%, J.T. Baker) at 60 °C for 48 h, followed by a heat treatment at 350 °C for 2.5 h in 95% Ar + 5% H_2_ to remove the PS microspheres completely. In this study, we also prepared conventional single-layer colloidal crystals and their inverse opals for comparison purposes. Table 2 lists the basic characteristics of PS microsphere suspensions and their corresponding parameters for electrophoresis of multilayer and single-layer colloidal crystals. 

### 2.3. Material Characterization 

The PS microsphere in different sizes were subjected to zeta potential measurements (Beckman Coulter, Delsa Nano C, Brea, CA, USA) to obtain their respective zeta potential. The pH values for PS colloidal suspensions were adjusted by the addition of minute amounts of 0.1 M HNO_3(aq)_ and NH_4_OH_(aq)_. The microstructures and morphologies of multilayer colloidal crystals and inverse opals with gradient pores were observed using a scanning electron microscope (SEM; Hitachi, SU-8010, Chiyoda City, Tokyo, Japan). The critical pore sizes (bottlenecks) within the inverse opals were determined by a capillary flow porometer (PMI, CFP-1200, Ithaca, NY, USA). For porometer analysis, the sample was detached from the ITO substrate as a free-standing inverse opaline film and cut to a circular shape of 1 cm diameter. The mechanical properties of inverse opals with gradient pores were determined by nano-indentation using a Bruker’s Hysitron TI 980 TriboIndenter with a Berkovich diamond indenter tip. The load was steadily increased to the maximum load of 10 mN and decreased to 0 mN at rate of 1 mN s^−1^. The penetration depth was kept below one-fifth of the corresponding sample thickness to minimize any substrate interference. At least 10 measurements were conducted for each sample and the resulting parameters, including hardness and reduced elastic modulus, were reported and discussed. 

## 3. Results and Discussion

### 3.1. Preparation of Multilayer Colloidal Crystals

**Figure 1:**
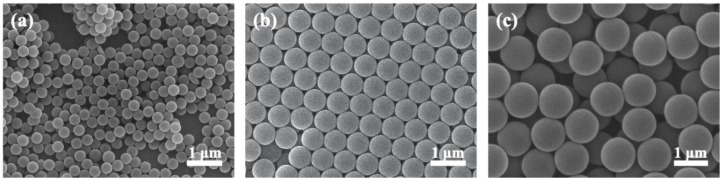
SEM images for PS microspheres with diameter of (**a**) 300, (**b**) 600, and (**c**) 1000 nm, respectively.

We recognized that the key to obtain desirable colloidal crystals is to synthesize monodispersive microspheres with appreciable surface charges. Unfortunately, the size distribution for commercially available PS microspheres is rather broad (ca. 10% standard deviation) and the magnitude of their surface charges vary widely, making it extremely difficult to construct large-area colloidal crystals with reduced crystallographic defects. Therefore, in our laboratory, we have developed the necessary synthetic steps to produce uniform PS microspheres with sufficient surface charges. In addition, our processing conditions could be fine-tuned to produce PS microspheres in different diameters. Figure 1 displays the SEM images of PS microspheres used in this study. Clearly, these PS microspheres adopted a spherical shape and their diameters were rather consistent.

**Figure 2:**
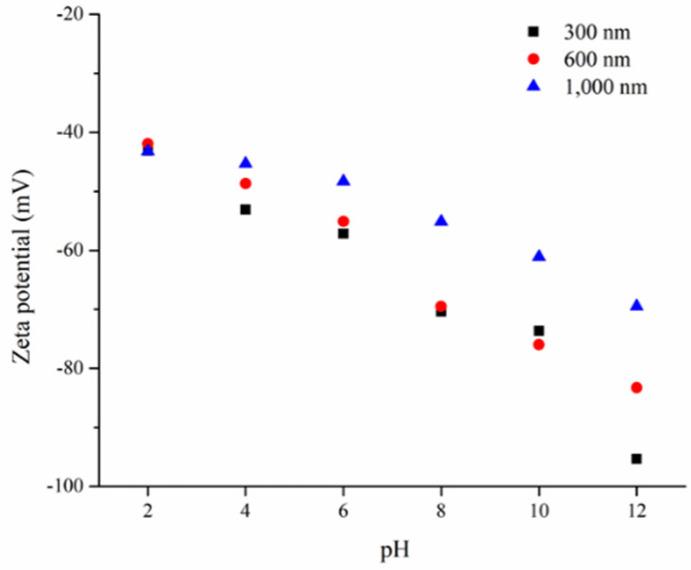
Zeta potentials for PS microspheres with diameter of 300, 600, and 1000 nm in a broad range of pH values.

Successful implementation of electrophoresis relies on a stable colloidal suspension so the zeta potential for the suspending colloids becomes critical. Figure 2 displays the respective zeta potentials for 300, 600, and 1000 nm PS microspheres in a broad range of pH values. Notably, a consistent pattern was observed showing a progressively negative zeta potential with increasing pH of the suspension. The PS microspheres are known to carry negative surface charges due to the decomposition of K_2_S_2_O_8_ and NaSS from the synthesis step that produces SO_4_^2−^ and HSO_3_^−^ ions re-adsorbing onto the PS microspheres. In an acidic solution, the PS microspheres are able to attract additional protons, rendering less negative surface charges. In contrast, in an alkaline solution, the adsorption of hydroxyl ions results in more negative surface charges. Interestingly, at identical pH, the 1000 nm microspheres revealed a consistently smaller negative zeta potential than those of 300 and 600 nm microspheres. This pattern was possibly due to its relatively smaller surface-to-volume ratio. In general, a zeta potential of ±40 mV is necessary for colloids to form a stable suspension, and a zeta potential greater than ±60 mV ensures a suspension with long-term stability. Therefore, our PS microspheres were able to form stable suspension for electrophoresis in the pH range of 2–12. 

Electrophoresis entails the migration of charged microspheres that are driven toward a substrate with opposite polarity. According to Besra and Liu, the deposition rate for electrophoresis is linearly proportional to the suspension concentration, electric field, and zeta potential [37]. In our laboratory, these parameters have been fine-tuned to obtain desirable single-layer colloidal crystals. However, to construct multilayer colloidal crystals with reduced defects, additional factors need to be considered and they are discussed as followed. 

#### 3.1.1. Size Effect

Multilayer colloidal crystals require PS microspheres in different sizes so their exact packing order matters. Our experiences indicate that larger microspheres could be deposited atop of smaller microspheres provided the latter ones are arranged with a uniform surface for larger microspheres to assemble on. Otherwise, if the microspheres are stacked in opposite way, the smaller microspheres during electrophoresis might be forced to squeeze into the interstitial voids and grain boundaries of the large microspheres underneath. As a result, the smaller microspheres are more likely to be randomly-packed at the interface, which severely impacts the crystallinity of top-layer colloidal crystals. 

#### 3.1.2. pH Effect

The pH of the suspension affects both zeta potential and mobility of PS microspheres in the suspension, as well as the lateral movements of microspheres on the substrate. Our experiences suggest that the first electrophoresis needs to be performed in suspension with slightly higher pH whereas the second electrophoresis requires a suspension with smaller pH. This is to ensure that the microspheres in the first-layer colloidal crystals are properly fixated in their respective positions to provide a stable interface for large microspheres to assemble on during the formation of second-layer colloidal crystals. 

#### 3.1.3. Electric Field

In electrophoresis, a stronger electric field leads to a faster deposition. In order to maintain the structural integrity of first-layer colloidal crystals, the second electrophoresis is carried out under a relatively stronger electric field. It is because a greater electric field helps in ‘gluing’ the microspheres in the first-layer colloidal crystals on the substrate. However, it is noted that the effect of increased electric field is opposite to that of lower pH of the suspension for second-layer colloidal crystals. Therefore, the exact magnitude of the electric field is carefully balanced during the second electrophoresis stage to minimize physical impacts of arriving microspheres on the crystallinity of first-layer colloidal crystals. 

#### 3.1.4. Solid Loading of PS Suspension

The above discussion focuses on the movement or mobility of individual PS microspheres and their effects on the crystallinity of colloidal crystals. In practice, the electrophoresis entails aggregated movements of many microspheres. Therefore, for the formation of multilayer colloidal crystals, the solid loadings for different PS suspensions need to be in a close range.

**Figure 3:**
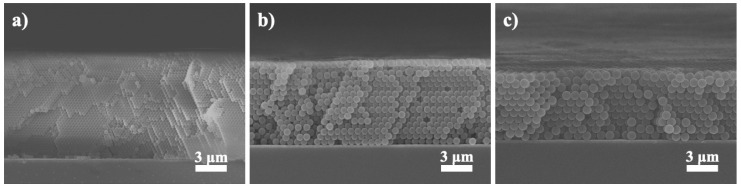
Cross-sectional SEM images for single-layer colloidal crystals assembled with PS microspheres of (**a**) 300, (**b**) 600, and (**c**) 1000 nm, respectively.

Figure 3a–c display the cross-sectional SEM images for single-layer colloidal crystals of 300, 600, and 1000 nm microspheres. As shown, they revealed hexagonal close-packed structures with impressive surface uniformity. Their respective thickness was 9.8, 8.2, and 7.7 µm, which corresponded to 46, 16, and 10 colloidal planes. Relevant electrophoresis parameters have been optimized and they are listed in Table 2. It is noted that from these images, the PS microspheres were properly stacked and colloidal grain boundaries were not observed.

**Figure 4:**
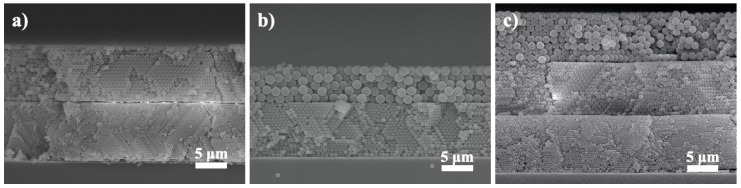
Cross-sectional SEM images for multilayer colloidal crystals assembled with PS microspheres of (**a**) 300/600, (**b**) 300/1000, and (**c**) 300/600/1000 nm respectively.

Figure 4a–c display the cross-sectional SEM images for multilayer colloidal crystals in different combinations of PS microspheres; 300/600 nm (Figure 4a), 300/1000 nm (Figure 4b), and 300/600/1000 nm (Figure 4c), respectively. Clearly, these multilayer colloidal crystals demonstrated rather similar structures as compared to their single-layer counterparts. Notably, the assembly of PS microspheres in different sizes retained a close-packed arrangement and their interface revealed a sharp and well-defined transition. These multilayer colloidal crystals maintained the structural integrity because the mild heat treatment we conducted effectively ‘glued’ the PS microspheres together. From our previous experiences, after electrophoresis a heat treatment at temperature below the glass transition temperature of PS microspheres (108 °C) caused slight deformation and better adhesion at the contact areas among neighboring PS microspheres. In addition, a slightly longer heat treatment time was necessary for multilayer colloidal crystals as it prevented the sample from disintegrating during subsequent electroplating process. 

### 3.2. Fabrication of Multilayer Inverse Opals

**Figure 5:**
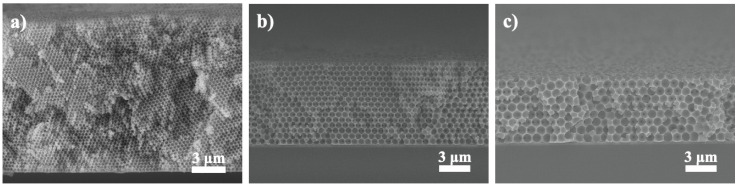
Cross-sectional SEM images for single-layer inverse opals of (**a**) 300, (**b**) 600, and (**c**) 900 nm, respectively.

Figure 5 displays the cross-sectional SEM images for single-layer Ni inverse opals of 300, 600, and 1000 nm PS microspheres. Apparently, the samples demonstrated an ideal honeycomb structure in which macropores were positioned in a hexagonal configuration. The macropores were formed by the removal of PS microspheres so their arrangements represented the close-packed structure of colloidal crystals. In addition, there were pore channels connecting neighboring macropores whose diameter was one-quarter to one-fifth of that of macropores. These interconnected pore channels arose from the contact areas between neighboring PS microspheres that prohibited the electroplating of Ni. In our experiences, the diameter of interconnected pore channels could be readily adjusted by the temperature and time of heat treatment after electrophoresis. In short, these SEM images confirmed that an ideal single-layer inverse opals was successfully produced. Their respective thickness was 13.2, 8.1, and 6.5 µm, representing 53, 16, and 10 inverse opaline layers. 

**Figure 6:**
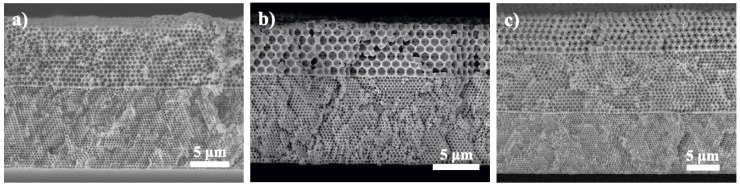
Cross-sectional SEM images for multilayer inverse opals of (**a**) 300/600, (**b**) 300/1000, and (**c**) 300/600/1000 nm, respectively.

Figure 6a–c display the cross-sectional SEM images for multilayer Ni inverse opals in different combinations of PS microspheres; 300/600 nm (Figure 6a), 300/1000 nm (Figure 6b), and 300/600/1000 nm (Figure 6c), respectively. The electroplating was performed properly with Ni growing successively from the bottom to the top as the surfaces of multilayer inverse opals were relatively flat without appearance of protruded islands and valleys. In addition, the order and size of macropores and interconnected pore channels were observed as expected. It is noted that the electroplating was kept at a modest rate to avoid any localized overplating, as residual stress was detrimental to the quality of colloidal crystals and inverse opals.

**Figure 7:**
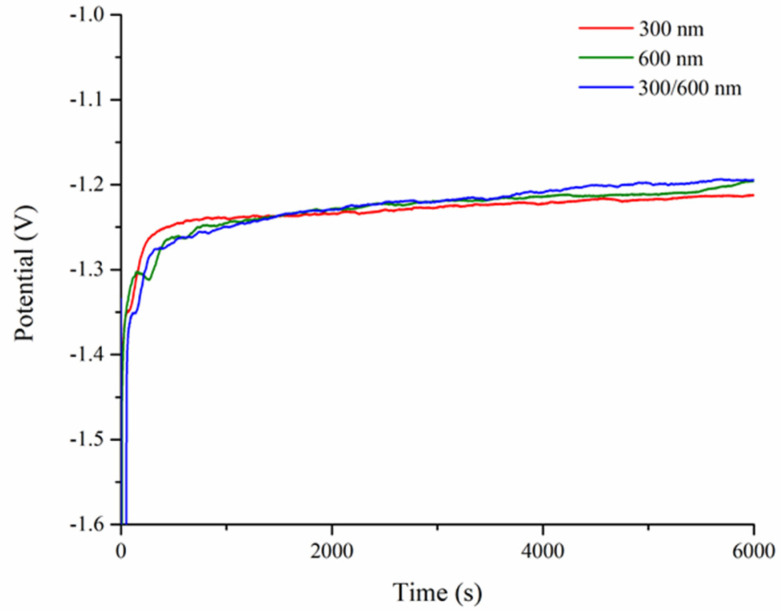
Voltage as a function of plating time for single-layer colloidal crystals of 300 and 600 nm, as well as multilayer inverse opals of 300/600 nm.

Despite the multilayer colloidal crystals being constructed with PS microspheres in different sizes, their close-packed structures in principle inferred a constant packing density of 74%, leaving 26% as the interstitial voids to be filled. Consequently, multilayer colloidal crystals consisted of different microspheres were expected to exhibit similar plating behaviors. Figure 7 displays the representative voltage profiles as a function of time during the galvanostatic plating of Ni for single-layer inverse opals of 300 and 600 nm, as well as multilayer inverse opals of 300/600 nm, respectively. It is noted that the magnitude of voltage recorded represented the electric resistance incurred during the electroplating process. As shown, among these three samples, their voltage profiles were rather similar; at the initial stage, the voltage started below −1.4 V and was reduced quickly to −1.27 V within the first 500 s, followed by a slow descent to −1.2 V at 6000 s. This behavior was consistent with what were reported earlier in similar experiments [35,38]. Since the Ni electrodeposition was conducted at a fixed current, the absolute value of the recorded voltage was proportional to the sum of electrolyte iR loss and charge transfer resistance of Ni^2+^/Ni. We recognized that the reduction of Ni^2+^ ions was straightforward so its charge transfer resistance was expected to be constant. Therefore, the observed reduction in voltage was associated with the decrease in electrolyte iR loss during the plating process. It is because, at the beginning, the reduction of Ni^2+^ took place at the bottom of colloidal crystals which represented the longest percolation length. As the Ni was filled up as time progressed, the electrolyte percolation distance became shorter, resulting in a smaller voltage. In short, from Figure 7, these similar voltage profiles inferred that the colloidal crystals were properly assembled, and the interface within the 300/600 nm multilayer colloidal crystals was continuous without revealing any abrupt voltage fluctuation.

### 3.3. Characterization of Multilayer Inverse Opals

**Figure 8:**
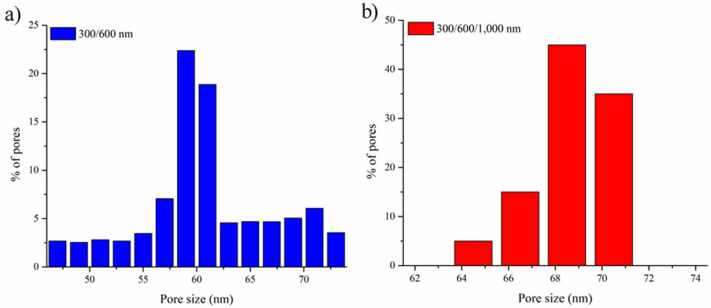
Bottleneck size distribution from PMI for free-standing multilayer inverse opaline films of (**a**) 300/600 and (**b**) 300/600/1000 nm, respectively.

The multilayer inverse opals demonstrated impressive structural robustness which enabled them to be detached easily from the ITO substrate, becoming essentially free-standing inverse opals with gradient pores. Therefore, it is feasible to determine the bottlenecks and their distributions using a porometer. In our samples, the bottlenecks were expected to be the diameter of interconnected pore channels residing within the inverse opals of smaller macropores, i.e., the bottom layer of multilayer inverse opals. For the sample of 300/600 nm, as shown in Figure 8a, the bottleneck was determined at 58–62 nm, which was around one-fifth of the macropore of 300 nm. Similarly, the bottleneck for the sample of 300/600/1000 nm revealed a size of 66–69 nm, as shown in Figure 8b. This slightly enlarged bottleneck from the sample of 300/600/1000 nm over that of 300/600 nm was due to their difference in the heat treatment time. It is because additional heat treatment led to more softening of the PS microspheres, and thus engendered larger interconnected pore channels. The results from PMI validated that the porous nature of multilayer inverse opals was identical to that of single-layer inverse opals, and sequential electrophoresis steps were properly performed to construct ideal multilayer colloidal crystals so their inverse opals with gradient pores were successfully fabricated. 

**Figure 9:**
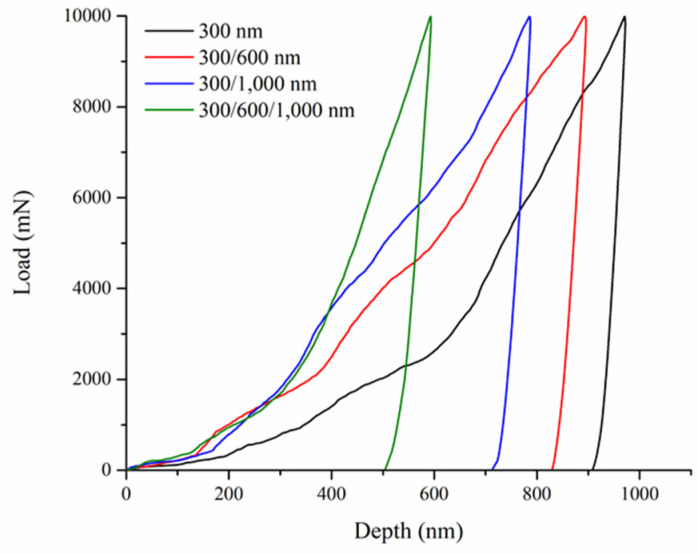
Load–displacement profiles for multilayer inverse opals of 300/600, 300/1000, and 300/600/1000 nm, as well as single-layer inverse opals of 300 nm.

In the literature, it has been established that the mechanical properties of nanostructured materials are affected by their pore sizes and spatial distribution [39,40,41]. To determine relevant mechanical properties of multilayer inverse opals, nanoindentation tests were conducted to obtain the load–displacement profiles. Figure 9 displays the load–displacement profiles for multilayer Ni inverse opals in different combinations; 300/600, 300/1000, and 300/600/1000 nm, as well as single-layer inverse opals of 300 nm for comparison purpose. Clearly, these load–displacement profiles were similar and our samples demonstrated sufficient mechanical strength to withstand the indentation without suffering from any visible fracturing. 

**Figure 10:**
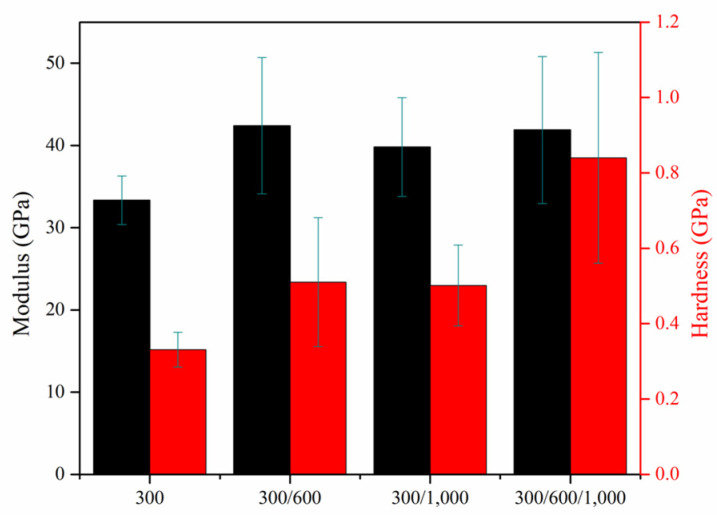
Bar charts for hardness and reduced elastic modulus of multilayer inverse opals of 300/600, 300/1000, and 300/600/1000 nm, as well as single-layer inverse opals of 300 nm.

Among these samples, the multilayer inverse opals revealed shallower indentation depths as compared to those of single-layer inverse opals, suggesting the robust nature of inverse opals with gradient pores. From these load–displacement profiles, both hardness (*H*) and reduced elastic modulus (*E_r_*) were derived using following equations [42].

(1) $$A\left(h_{c}\right)=24.5h_{c}{}^{2}$$

(2) $$H=\frac{P_{max}}{A}$$

(3) $$E_{r}=\frac{1}{2}\frac{dP}{dh}\sqrt{\frac{\pi }{A}}$$

where the *A* is the projected area of the elastic contact, the *h_c_* is the contact depth, the *P_max_* is the maximum load upon loading, and the $\frac{dP}{dh}$ is the experimental stiffness of the unloading data. Figure 10 displays the bar charts of hardness and reduced elastic modulus for multilayer Ni inverse opals in different combinations; 300/600, 300/1000, and 300/600/1000 nm, as well as single-layer inverse opals of 300 nm. As shown, the hardness for multilayer inverse opals was consistently greater than that of single-layer counterpart. In particular, the inverse opals of 300/600/1000 nm revealed the largest hardness (0.84 GPa) whereas the inverse opals of 300/600 and 300/1000 nm exhibited similar hardness values (~0.51 GPa). These represented 154% and 55% improvements over that of single-layer inverse opals (0.33 GPa). Our results indicated that inverse opals with gradient pores were able to sustain stronger external stress. We surmised that in multilayer inverse opals, individual inverse opaline grains were effectively confined within their respective single-layer structures, and their orientations were presumed to be random. In contrast, in single-layer inverse opals, the growth of inverse opaline grains was unrestricted and was expected to extend vertically from the bottom to the top, resulting in grains with larger physical sizes. Therefore, our observation of stronger hardness in multilayer inverse opals is most likely to be similar to the ‘grain size’ effect in physical metallurgy in which smaller grains provide more grain boundaries to inhibit the movement of dislocations, leading to a stronger mechanical strength. In contrast, for reduced elastic modulus, there appeared moderate improvements for the multilayer inverse opals over that of single-layer counterpart. It is because the magnitude of reduced elastic modulus is associated with the interatomic bonding strength and the defective states of inverse opaline skeletons. In principle, their values are expected to be relatively unchanged between single-layer and multilayer inverse opals. 

## 4. Conclusions

In short, we successfully demonstrated the fabrication of free-standing inverse opals with gradient pores and reported their improved mechanical properties. We recognize that the successful preparation of multilayer colloidal crystals with different combinations of constituting microspheres (size and type) makes it feasible to form multilayer inverse opals with many possible new applications. We adopted a sequential electrophoresis technique to prepare multilayer colloidal crystals consisted of PS microspheres in size of 300, 600, and 1000 nm, respectively. The electrophoresis parameters were optimized so the larger PS microspheres were stacked atop of smaller ones in an orderly manner. The inverse opals with gradient pores were prepared by galvanostatic plating of Ni within the colloidal template, followed by the selective removal of PS microspheres. The SEM images exhibited ideal close-packed structures with well-defined interfaces among different layers. Results from PMI revealed a size of bottlenecks consistent with those of interconnected pore channels. Mechanical properties determined by nanoindentation tests indicated significant enhancement in hardness and moderate improvement in reduced elastic modulus from multilayer inverse opals as compared to those of single-layer ones. The increased mechanical stability favors further development of these nanostructured materials in practical applications, such as their direct utilization or the leverage in support materials.

## Figures and Tables

**Table 1:** Chemicals used in synthesizing PS microspheres in different parameters

PS Microspheres ID	St (g)	NaSS (mg)	KHCO_3_ (mg)	Average Size (Mean ± Std. Dev., nm)
300 nm	160	500	200	315 ± 12
600 nm	160	10	200	620 ± 16
1000 nm	160	0.5	200	1025 ± 23

**Table 2:** Specifications for colloidal suspensions, as well as the strength of electric field and time during electrophoresis for multilayer colloidal crystals

PS Microspheres ID	wt %	pH	Zeta Potential (Mean ± Std. Dev, mV)	E-Field (V cm^−1^)	EPD Time (min)
300 nm	0.8	9	−71.2 ± 2.2	6	10
600 nm	0.8	7.5	−63.6 ± 0.8	7	10
1000 nm	1	7	−53.5 ± 1.4	10	10
